# *Stenotrophomonas maltophilia* Bloodstream Infection Outbreak in Acute Care Hospital, California, USA, 2022–2023^1^

**DOI:** 10.3201/eid3203.250835

**Published:** 2026-03

**Authors:** Sana M. Khan, Axel A. Vazquez Deida, Steven Langerman, Jennifer C. Hunter, Rebeca Elliott, Alison Laufer Halpin, Alyssa G. Kent, Paige Gable, Heather A. Moulton-Meissner, Frances C. Knight, Thomas Ewing, Kristen Clancy, Amit Chitnis, Eileen F. Dunne, Dustin Heaton, Barbara Allen, Hillary Metcalf, Munira Shemsu, Kathleen Nava, Suada Abdic, Kiran M. Perkins, Elsa Villarino, Jeffrey Silvers, Kavita K. Trivedi

**Affiliations:** Centers for Disease Control and Prevention, Atlanta, Georgia, USA (S.M. Khan, A.A. Vazquez Deida, S. Langerman, J.C. Hunter, A. Laufer Halpin, A.G. Kent, P. Gable, H.A. Moulton-Meissner, F.C. Knight, T. Ewing, K. Clancy, K.M. Perkins); Alameda County Public Health Department, San Leandro, California, USA (S.M. Khan, A. Chitnis, E.F. Dunne, D. Heaton, M. Shemsu, K.K. Trivedi); California Department of Public Health, Richmond, California, USA (R. Elliott, B. Allen, H. Metcalf, E. Villarino); Sutter Health, Sacramento, California, USA (K. Nava, S. Abdic, J. Silvers)

**Keywords:** *Stenotrophomonas maltophilia*, bacteria, outbreak, healthcare-associated infections, case-control study, California, United States

## Abstract

*Stenotrophomonas maltophilia* is an opportunistic bacterial pathogen found in healthcare settings. During May 2022–September 2023, an acute care hospital in northern California, USA, identified 13 *S. maltophilia* bloodstream infections among intensive care unit patients. Whole-genome sequencing showed the isolates were highly related. We identified risk factors for infection by conducting a matched case–control study, targeted assessment of infection prevention and control practices, and laboratory testing of suspected environmental reservoirs. Among 13 case-patients and 39 control-patients, patients exposed to iodinated contrast (odds ratio [OR] 12.0; 95% CI 2.1–∞), injectable propofol (OR 12.2; 95% CI 1.5–101.4), or fentanyl (OR 9.2; 95% CI 1.8–∞) had increased odds of *S. maltophilia* bloodstream infection. Although we did not have culture confirmation of a source, we suspect *S. maltophilia* was transmitted by exposure to nonsterile water from a common source. We recommended infection prevention and control practices to reduce risk for contamination from nonsterile water.

*Stenotrophomonas maltophilia* is a gram-negative bacterium found in aqueous environments, including hospital water systems. *S. maltophilia* causes opportunistic healthcare-associated infections that can result in increased illness and death among hospitalized patients, especially critically ill or immunocompromised patients (*1*,*2*). *S. maltophilia* often leads to respiratory tract infection but can also cause bloodstream, intraabdominal, urinary, catheter, and implanted device–associated infections (*2*,*3*). The crude mortality rate for *S. maltophilia* bloodstream infections (BSIs) ranges from 14% to 69% (*2*,*4*–*6*). Risk factors for healthcare-associated *S. maltophilia* bacteremia include indwelling devices, prior antimicrobial drug therapy, and prolonged hospitalization (*1*,*7*). Past outbreaks of *S. maltophilia* infections in healthcare settings have been attributed to exposure to nonsterile water and contaminated medical devices, medications, or patient care products (*8*–*10*). *S. maltophilia* infections are particularly concerning because the bacterium is often resistant to multiple classes of antibiotics, and treatment options are limited (*2*,*11*).

In May 2022, an acute care hospital in California, USA, alerted its local public health department about 2 patients with *S. maltophilia* BSIs in an intensive care unit (ICU). The hospital identified a third case in July 2022 and reported it to the local health department, after which the hospital and local health department consulted the California Department of Health (CDPH). The hospital infection control team identified an additional 6 *S. maltophilia* BSIs during July–September 2022 at the same ICU. The local health department and CDPH consulted the Division of Healthcare Quality Promotion (DHQP), National Center for Emerging and Zoonotic Infectious Diseases, Centers for Disease Control and Prevention (CDC; Atlanta, Georgia), on August 22, 2022, for technical assistance, including whole-genome sequencing (WGS). WGS on 6 available patient isolates showed that all isolates were sequence type (ST) 239 and highly related. No source for the infections was identified at that time, but CDPH and the local health department provided infection control recommendations to the facility during on-site visits in 2022. After a 6-month lapse in cases, the facility reported 4 additional *S. maltophilia* BSIs during April–September 2023, and DHQP provided onsite assistance during August 28–September 12, 2023. We describe the initial and follow-up investigations in response to *S. maltophilia* BSIs.

## Methods

### Epidemiologic Investigation

We conducted a matched case–control study to determine risk factors for *S. maltophilia* BSI. We identified case-patients via *S. maltophilia*–positive blood cultures. After the initial case was identified in May 2022, we subsequently identified cases by using blood and respiratory cultures from hospital inpatients who had *S. maltophilia* isolated. We defined a case-patient as a febrile (temperature >38°C) ICU patient with *S. maltophilia* isolated from a blood culture during May 2022–September 2023.

For each case-patient, we assessed exposures during a reference period, which we defined as the number of days from hospital admission to index specimen collection. The index specimen was the first specimen from which *S. maltophilia* was isolated for a given case-patient. We selected 3 control-patients for each case-patient by matching the closest hospital admission in calendar time and an ICU stay greater than or equal to the matched case-patient’s reference period.

We abstracted patient information and relevant healthcare exposures from electronic medical records. For case-patients, we abstracted exposures during the reference period; for control-patients, we abstracted information on the basis of their matched case-patient’s reference period. For example, if a case-patient had 5 days between date of admission and date of index specimen collection, we abstracted exposures for 5 days after admission for the matched control-patients. Abstracted information included demographic characteristics, procedures conducted, admission location, medications administered, and any indwelling medical devices (e.g., central venous catheters or urinary catheters placed during admission). Clinical and laboratory data collected included admission diagnoses and microbiology results. 

This activity was defined by CDC as a public health investigation and therefore institutional review board approval was required. This activity was reviewed by CDC and was consistent with applicable federal law and CDC policy (see e.g., 45 C.F.R. part 46.102(l)(2), 21 C.F.R. part 56; 42 U.S.C. §241(d); 5 U.S.C. §552a; 44 U.S.C. §3501 et seq.).

### Statistical Analysis

We summarized patient characteristics by using descriptive statistics and used univariate conditional logistic regression to estimate the unadjusted odds ratio (OR) and 95% CIs for each possible risk factor, such as exposure to any imaging and invasive procedures, injectable medications received, and presence of indwelling devices. We used exact conditional logistic regression for characteristics in which all case-patients or all control-patients had the exposure of interest. We performed statistical analysis by using SAS version 9.4 (SAS Institute Inc., https://www.sas.com).

### Onsite Infection Control Assessment

During the 2022 and 2023 site visits, we observed infection prevention and control (IPC) practices in patient care areas where most case-patients received care, including the emergency department (ED), ICU, ICU step-down unit, and interventional radiology (IR) and computed tomography (CT) suites. We also observed environmental cleaning and disinfection practices. In addition to direct observation of IPC practices, we conducted informal interviews with staff in each department. In 2023, we additionally observed bedside blood draws, trauma patient intake, IR and CT procedures, and maintenance of the contrast injection system in the CT suite.

The facility conducted a review of case-patient hospital stays to assess whether drug diversion occurred. Staff reviewed relevant activity documented in the electronic health records and the automated medication dispensing systems from the time of admission through index culture collection for the affected patients.

### Environmental Sampling, WGS, and Bioinformatic Analysis

We conducted 2 rounds of environmental sampling. During August–October 2022, we collected water and swab samples from sink drains and faucets from the ED, operating rooms, radiology suites, and certain case-patient rooms in the ICU. In September 2023, we collected environmental and product samples informed by direct observation and epidemiologic hypotheses for exposure on the basis of review of preliminary statistical analysis of potential risk factors. We also collected samples from the ED critical care bay, the CT suite, and the ICU, including swabs of sink drains, sponge-wipes used on the contrast autoinjector, and first-catch water samples from 2 sinks in the ED and both CT suite rooms. We obtained samples from the contrast autoinjector in each CT room by using a swab and sponge-wipe. We also obtained materials used during the autoinjector’s maintenance for sampling, such as towels used to clean the machine.

We evaluated water samples for *S. maltophilia* by concentration with membrane filtration standard methods (*12*). We filtered 10 mL, 100 mL, and 200 mL of water through a 0.45-micron pore filter (Pall Corporation, https://www.pall.com) and placed filtered samples onto MacConkey agar (MAC; BD Biosciences, https://www.bdbiosciences.com) or directly into 30-mL of tryptic soy broth (TSB; Hardy Diagnostics, http://www.hardydiagnostics.com). We separated sponge-wipes from their handles before processing by using neutralizing buffer (Neogen, https://www.neogen.com), then homogenized at 200 rpm in a Stomacher Circulator 400C (Seward, https://www.seward.co.uk) in phosphate-buffered saline containing 0.02% Tween 80. We then removed the sponge portion and concentrated the homogenate by centrifugation at 2,700 × *g* for 20 minutes. We discarded most of the supernatant and resuspended the pellet in the residual supernatant, which was ≈5 mL. We then plated 100 µL of the concentrated eluent onto MAC and trypticase soy agar with 5% sheep blood (BAP; BD Biosciences) and placed the remaining eluent in 10 mL of TSB. We placed amies transport media swabs directly into 10 mL of TSB. We incubated all broths and culture agar plates for 18–24 hours at 35°C. After incubation, we plated turbid broth enrichments onto MAC and BAP agar and incubated for 18–24 hours at 35°C. We screened all plate cultures for suspected *S. maltophilia*. We used MALDI Biotyper (Bruker Daltonics, https://www.bruker.com) matrix-associated laser desorption/ionization time-of-flight and CDC’s MicrobeNet (https://microbenet.cdc.gov) databases to identify suspected *S. maltophilia* and considered scores >2.0 confident for species-level identification.

Of 13 case-patients, 10 (77%) had >1 clinical blood isolate with *S. maltophilia* growth available for WGS. We also collected 8 clinical respiratory isolates with *S. maltophilia* growth from non–case-patients for WGS. We used Maxwell 16 Cell with Low Elution volume DNA Purification Kit, (both Promega, https://www.promega.com) to extracted DNA from pure isolates and subsequently sheared DNA by using the ME200 Focused-Ultrasonicator (Covaris, https://www.covaris.com). We prepared indexed libraries on the Zephyr G3 NGS workstation (PerkinElmer https://www.perkinelmer.com) by using Ovation Ultralow System V2 Kit (Tecan Life Sciences, https://lifesciences.tecan.com) and analyzed on the Fragment Analyzer System with Standard Sensitivity NGS Fragment Analysis Kit (both Agilent, https://www.agilent.com). We performed WGS on the MiSeq sequencer by using the MiSeq Reagent Kit v2 (Illumina, https://www.illumina.com), which generated 2 × 250 paired-end reads.

We processed raw sequencing reads through DHQP’s in-house QuAISAR-H pipeline (https://github.com/DHQP/QuAISAR_singularity). Specifically, we processed sequences by using BBDuk version 38.90 (https://jgi.doe.gov/data-and-tools/bbtools) for adaptor and PhiX removal, and fastp version 0.20.1 to quality trim sequences (*13*). We performed de novo assembly of samples by using SPAdes version 3.15.0 (*14*). After assembly, we removed small scaffolds (<500 bp). We used the PubMLST database and mlst version 2.19 (*15*) to assign STs. We generated maximum-likelihood phylogenetic trees from high-quality single-nucleotide variant (hqSNV) alignments by using SNVPhyl–Nextflow version 1.0.0 to assess isolate relatedness (*16*). We also ran identified clusters separately to calculate cluster hqSNV distances and core-genome estimates. For all SNVPhyl analyses, the reference was the centroid of the isolate set determined by using mash version 2.0 b (*17*). We deposited sequences in the National Center for Biotechnology Information’s Sequence Read Archive (BioProject PRJNA288601, BioSample nos. SAMN45204371–413).

## Results

### Epidemiologic Investigation

**Figure 1:**
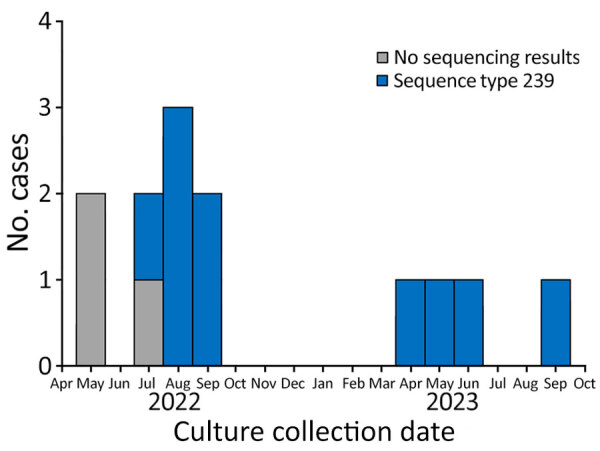
Number of cases per month in *Stenotrophomonas maltophilia* bloodstream infection outbreak in acute care hospital, California, USA, 2022–2023. Graph includes isolates with whole-genome sequencing results showing sequence type 239.

We identified 13 case-patients who were hospitalized during May 2022–September 2023 (Figure 1); we selected 39 control-patients for matching. After the initial case was identified in May 2022, blood and respiratory isolates for all hospital inpatients were assessed for *S. maltophilia* growth; no *S. maltophilia* BSIs were identified in patients outside the ICU. Case-patients had a lower median age, 45 (IQR 36.0–68.0) years, than control-patients, who had a median age of 64.0 (IQR 60.0–75.0) years (Table 1). The median reference period was 5 (IQR 3–10) days.

**Table 1:** Characteristics of case-patients and control patients in a *Stenotrophomonas maltophilia* bloodstream infection outbreak in acute care hospital, California, USA, 2022–2023*

Characteristics	No. (%) case-patients, n = 13	No. (%) control-patients, n = 39
Median age, y (IQR)	45 (36.0–68.0)	64.0 (60.0–75.0)
Sex		
M	9 (69.2)	27 (69.2)
F	4 (30.8)	12 (30.8)
Race		
White	5 (46.2)	14 (35.9)
Black/African American	2 (15.4)	9 (23.1)
Asian	1 (7.7)	8 (20.5)
American Indian/Alaska Native	1 (7.7)	0
Native Hawaiian or other Pacific Islander	0	2 (5.1)
Other	4 (23.1)	6 (15.4)
Ethnicity		
Hispanic	3 (23.0)	8 (22.2)
Non-Hispanic	10 (77.0)	28 (77.8)
No. underlying conditions†		
0	6 (46.2)	9 (23.1)
1	6 (46.2)	13 (33.3)
2	1 (7.7)	10 (25.6)
3	0	4 (10.3)
4	0	3 (7.7)
Admitted to ICU from ED	12 (92.3)	36 (92.3)
Critical care bay of ED	10 (76.9)	26 (66.7)
On immunosuppressant treatments	1 (7.7)	3 (7.7)
Median no. (IQR) days between admission and positive culture collection	5 (3–10)	NA

*ED, emergency department; ICU, intensive care unit; NA, not applicable. †Underlying conditions were assessed by using electronic health records and included coronary artery disease, congestive heart failure, diabetes, peripheral vascular disease, gastrointestinal disease, liver disease, chronic kidney disease, obesity, cancer, and hypertension.

Most (92.3%, 48/52) patients were admitted to the ICU from the ED. A larger percentage (76.9%, 10/13) of case-patients than control-patients (66.7%, 26/39) were admitted through the critical care bay in the ED (Table 2). All 13 case-patients received iodinated contrast, either through an IR procedure or CT scan, compared with only 64.1% (25/39) control-patients. In addition, all 13 case-patients were exposed to injectable fentanyl and 12 (92.3%) were exposed to injectable propofol, but for control-patients, only 64.1% (25/39) were exposed to injectable fentanyl and 61.5% (24/39) to propofol. Case-patients and control-patients had similar rates of antimicrobial drug exposure (84.6% [11/13] vs. 82.1% [32/39]).

**Table 2:** Conditional logistic regression models on risk factors of interest for a *Stenotrophomonas maltophilia* bloodstream infection outbreak in acute care hospital, California, USA, 2022–2023*

Risk factors	No. (%) case-patients, n = 13	No. (%) control-patients, n = 39	OR (95% CI)
Imaging procedures			
Any CT†‡	13 (100)	37 (94.9)	0.81 (0.10–∞)
CT with contrast	11 (91.7)	23 (63.9)	8.5 (0.9–78.2)
Ultrasound	8 (61.5)	23 (59.0)	1.1 (0.3–4.5)
MRI	1 (7.7)	9 (23.1)	0.3 (0.03–2.3)
Injectable medications			
Exposure to any contrast†§	13 (100)	25 (64.1)	12.0 (2.1–∞)
Fentanyl†*	13 (100)	24 (61.5)	9.2 (1.8–∞)
Propofol	12 (92.3)	19 (48.7)	12.2 (1.5–101.4)
Famotidine	9 (69.2)	29 (74.4)	0.8 (0.2–3.0)
Locations			
CT room 2	9 (69.2)	15 (38.5)	3.6 (0.9–14.7)
CT room 1	8 (61.5)	23 (59.0)	1.1 (0.3–3.6)
Critical care bay	10 (76.9)	26 (66.7)	1.6 (0.4–6.9)
Procedures			
Any procedure¶	10 (76.9)	22 (56.4)	2.6 (0.6–10.5)
Surgery	7 (53.9)	12 (30.8)	2.6 (0.7–9.7)
Any transfusions	7 (53.9)	15 (38.5)	1.9 (0.5–7.1)
Operating room procedure	6 (46.2)	12 (30.8)	1.9 (0.5–6.8)
Interventional radiology procedure	4 (30.8)	12 (30.8)	1.0 (0.2–4.2)
Bedside procedure	4 (30.8)	2 (5.1)	10.2 (1.1–93.0)
Indwelling devices			
Any tube#	12 (92.3)	30 (76.9)	3.3 (0.4–28.3)
Peripheral line	12 (92.3)	36 (92.3)	1.0 (0.06–16.0)
Mechanical ventilator	10 (76.9)	28 (71.8)	1.3 (0.3–6.2)
Arterial line	9 (69.2)	13 (33.3)	4.1 (1.1–16.2)
Central venous catheter	8 (61.5)	22 (56.4)	1.2 (0.4–4.3)
Other indwelling device**	7 (53.9)	36 (92.3)	0.12 (0.03–0.6)
Other factors			
Presence of wound	6 (46.2)	20 (51.3)	0.8 (0.2–2.9)
Antibiotic drug exposure	11 (84.6)	32 (82.1)	1.2 (0.2–7.0)

*CT, computed tomography; MRI, magnetic resonance imaging; OR, odds ratio. †Exact odds ratio; median unbiased estimate and 1-sided p value. ‡Brain, chest, abdomen, pelvis, maxillofacial, spine, head and neck, and other CT imaging. §Contrast administered during CT or other interventional radiologic procedure, ¶Procedures were surgery, transfusions, procedures in an operating room, interventional radiology, or bedside procedures. #Nasogastric, orogastric, percutaneous endoscopic gastronomy, and other tubes. **Suprapubic catheter, Foley catheter, dialysis line, surgical drain, or other devices.

### Statistical Analysis

A conditional logistic regression analysis revealed that case status was significantly associated with exposure to iodinated contrast either through CT imaging or an IR procedure (OR 12.0 [95% CI 2.1–∞]) (Table 2). Case status was also significantly associated with exposure to injectable fentanyl (OR 9.2 [95% CI 1.8–∞]) or propofol (OR 12.2 [95% CI 1.5–101.4]). Other risk factors associated with being a case-patient included having a bedside procedure (OR 10.2 [95% CI 1.1–93.0]) or having an arterial line (OR 4.1 [95% CI 1.1–16.2]). However, neither of those risk factors had >70% exposure among case-patients.

### Onsite Infection Control Assessment

We identified opportunities for improved IPC practices in certain hospital areas, including the CT suite and ICU, during our 2022 site visits. We conducted additional onsite IPC assessments in July, August, and September 2023, in response to identifying additional case-patients. Interviews with staff revealed that nonsterile tap water was used extensively to clean the contrast autoinjector, particularly to clean spilled iodinated contrast on and within the autoinjector. Service records for the CT autoinjectors indicated device exposure to tap water in the past, including an incident where standing water was discovered within one of the devices. We reviewed the manufacturer’s instructions for use (IFU), which state, “Do not use strong cleaning agents and solvents. Warm water and a mild disinfectant are all that are required to clean the [...] module” (*18*). The facility followed the manufacturer’s guidelines and, therefore, followed proper infection prevention protocols. The facility recommended that, in the CT suite, the staff use sterile water and not tap water for cleaning the contrast injection system to prevent possible contamination of the autoinjector with water-associated organisms, such as *S. maltophilia*, even though the use of warm tap water is allowed for cleaning per the manufacturer’s IFU.

In reviewing case-patient hospital stays to assess for drug diversion, the facility’s drug diversion committee did not find any evidence of drug diversion. The committee reviewed case-patient electronic health records and the automated medication dispensing systems from the time of admission through the culture collection for the affected patients and found no staff commonalities that would suggest drug diversion.

### Environmental Sampling, WGS, and Bioinformatic Analysis

**Figure 2:**
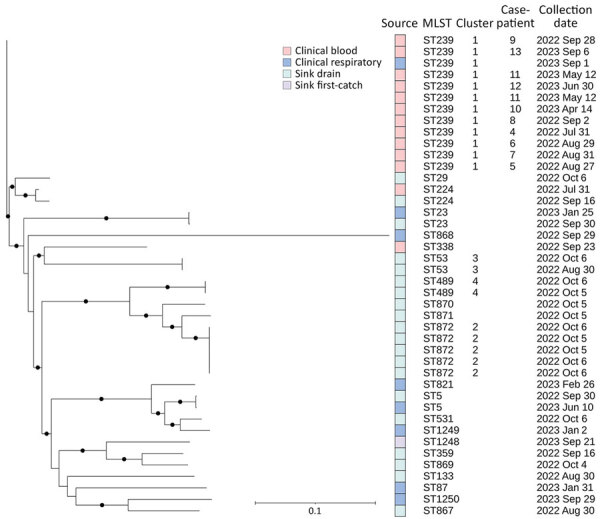
Phylogeny of *Stenotrophomonas maltophilia* detected in bloodstream infection outbreak in acute care hospital, California, United States, 2022–2023. Case-patient numbering starts at 4 because isolates for the first 3 cases were unavailable for sequencing. Full circles on the phylogenetic tree indicate evolutionary branching in the tree with a high value (n = 1) or strong support for the hypothesis that the branching is true. Cluster 1 (n = 12) differed by 0–4 pairwise high-quality single-nucleotide variants (hqSNVs), across 98.54% of the reference isolate core-genome. Cluster 2 (n = 5) differed by 1–21 hqSNVs, across 97.56% of the core-genome. Cluster 3 (n = 2) differed by 12 hqSNVs, across 98.68% of the core-genome. Cluster 4 (n = 2) differed by 3 hqSNVs, across 99.01% of the core-genome. Scale bar indicates nucleotide substitutions per site.

Environmental samples from the CT autoinjectors and ICU patient room sink yielded no growth for any gram-negative organisms. *S. maltophilia* was isolated from multiple sink drains and from a first-catch water sample collected from the sink in one of the CT rooms, but those isolates were not genetically linked to the outbreak strain (Figure 2). We sequenced 42 *S. maltophilia* isolates, including 13 (31%) blood, 8 (19%) respiratory, and 21 (50%) environmental samples (Figure 2). The 11 clinical blood isolates available for sequencing from 10 case-patients were highly related. 

We sequenced 2 additional blood isolates from 2 non–case-patients who had *S. maltophilia* bacteremia at hospital admission. Those 2 isolates were not genetically linked to each other or to the outbreak strain. Of 8 clinical respiratory isolates collected from non–case-patients during the same period, most (n = 7) did not match the *S. maltophilia* outbreak strain. 

Our analyses showed 4 distinct *S. maltophilia* clusters among the 42 isolates. Cluster 1 comprised 12 clinical isolates (11 from blood); all were ST239 and differed by 0–4 pairwise hqSNVs across 98.55% of the reference core genome. Clusters 2–4 included only environmental isolates (no clinical isolates) and included highly related isolates (cluster 2 hqSNV range 1–21, cluster 3 hqSNV difference 12, and cluster 4 hqSNV difference 2) from different sampling locations. The other isolates represented unique sequence types, except for 2 ST5, 2 ST23, and 2 ST224 isolates; isolates in those pairs were unrelated by phylogenetic analyses despite being the same sequence type.

## Discussion

This outbreak was unusual in the number of patients with BSIs with the same ST239 *S. maltophilia* identified during an 18-month period, including a 6-month interval without any cases. Prior to the outbreak period, only 1 *S. maltophilia* BSI was identified at this acute care hospital since 2020. Although we did not identify a single common source, *S. maltophilia* was likely transmitted through exposure to tap water through medications, medical devices, or patient care items. *S. maltophilia* is a biofilm-forming pathogen and might exist in a biofilm within the hospital’s water drain system despite the facility’s robust water management program. Nonsterile water might have contaminated patient care items, injectable contrast, or other medications that were used or prepared in a sink’s splash zone area. However, patients were hospitalized in multiple different rooms on different wings of the ICU and water sampling did not identify the *S. maltophilia* outbreak strain. ICU rooms had free-standing sinks without counter tops, making exposure of patient care items to tap water less likely in the ICU. Both rooms in the CT suite had sinks where injectable contrast or other medications could have been prepared for administration, and both sinks had attached countertops and no splash guards in place. 

*S. maltophilia* BSIs most often affect immunocompromised patients and have been associated with ICU admission, indwelling devices, and prior antibiotic use (*3*,*19*,*20*). *S. maltophilia* often leads to infection of the respiratory tract but can also cause BSIs through hematogenous dissemination from the respiratory tract (*21*). This outbreak was unusual in that the case-patients exhibited signs of *S. maltophilia* BSI without a prior or concomitant *S. maltophilia* respiratory infection. In addition, antibiotic exposures were of short duration and were similar between case- and control-patients. Of note, the ST239 *S. maltophilia* strain implicated in this outbreak seemed to have unusually low virulence because no case-patients developed sepsis or died from their bacteremia. In addition, most (7/8) clinical respiratory isolates collected from non–case-patients during the same period did not match the outbreak strain of *S. maltophilia*. One clinical respiratory isolate in September 2023 did match the *S. maltophilia* outbreak strain. That patient was not considered a case-patient because the patient did not have a BSI and thus did not meet the case definition for this outbreak investigation. However, a review of that patient’s medical chart revealed that the patient underwent a CT with contrast, was admitted to the ICU, and was exposed to both fentanyl and propofol between the time of admission and *S. maltophilia* culture collection date. That finding might be explained by opportunities for improvement of IPC practices that might have exposed the patient’s respiratory tract to the same unidentified nonsterile water source.

Although we isolated *S. maltophilia* from multiple water and sink drain samples, none of those isolates were related to the outbreak strain by WGS. In addition, sampling of the 2 CT autoinjectors did not reveal *S. maltophilia* growth. However, our observations of cleaning and maintenance practices revealed opportunities for potential contamination of the autoinjectors with nonsterile water. Previous studies have demonstrated that rinsing medical equipment with nonsterile water during cleaning can result in contamination of equipment and lead to subsequent BSIs among patients (*22*). In the setting of this outbreak, we recommended that the facility continue to identify opportunities to minimize the use of nonsterile water during maintenance and routine cleaning of the equipment to prevent additional *S. maltophilia* BSIs and other gram-negative bacterial infections. Since the outbreak, the facility has revised internal preventive maintenance guidelines for CT autoinjectors to include use of sterile water in place of the manufacturer’s IFU that states warm tap water may be used. In addition, staff working in the CT suite have implemented a daily deep cleaning of the injector heads using hospital-approved disinfectant wipes and a contrast cleaner for cleaning drips or spills. A 2-person approach was also implemented to eliminate use of the countertop next to the sink during the preparation and administration of contrast.

Several outbreaks, including ones involving bacterial infections, have been reported in healthcare settings as a result of drug diversion; some outbreaks involved tampering with injectable fentanyl (*23*). Drug diversion occurs when a prescription drug is removed from its intended path from the manufacturer to the patient. A 2015 review of the published literature and internal CDC records related to infections from drug diversion found 6 outbreaks over a 10-year period, 4 of which involved tampering with syringes or vials containing fentanyl (*23*). Tampering with syringes can include replacement of medication with saline or nonsterile water, enabling introduction of environmental pathogens to the bloodstream. Dilution of analgesic medication using nonsterile water has also been linked to an outbreak of *Sphingomonas paucimobilis*, another water-associated pathogen that rarely causes bloodstream infections (*24*). We found that exposure to fentanyl increased the odds of being a case-patient by 9.2 times and exposure to propofol increased the odds by 12.2 times. However, the facility’s drug diversion committee did not find evidence of drug diversion related to this outbreak. The review did not reveal any patterns or commonalities among the case-patients, such as a common anesthesia provider or nursing staff. The facility also contacted the contracted pharmacies and confirmed no medication recalls had been issued to ensure no intrinsic contamination of intravenous medications occurred during this time.

Although other risk factors were statistically associated with being a case-patient in this case–control study, we focused our investigation and discussion on iodinated contrast and injectable fentanyl, because almost all case-patients were exposed those risk factors. The possibility that the infection point source was a risk factor that was not common among case-patients is less likely.

The first limitation of this investigation is that, because of technical difficulties during environmental sampling, only 1 of 2 autoinjectors from the CT suite could be sampled initially. The local public health department returned to the facility on September 29, 2023, to sample the second autoinjector and the drain in the second CT room. *S. maltophilia* was not isolated from those samples, which might have been attributable to a 5-day delay between sample collection and culturing caused by shipping disruption between sample collection and receipt by the CDC laboratory. Potential temperature deviations during delayed shipping might have contributed to loss of organism viability. Second, biofilms are dynamic in nature, and the environmental samples collected might have been insufficient to capture a portion of the biofilm that contained the pathogen of interest (*25*). The negative environmental samples do not definitively rule out the presence of the *S. maltophilia* outbreak strain in the sampled environments. Despite those challenges, the matched case–control study and IPC observations provided the facility with focus areas for re-educating staff and auditing IPC practices surrounding using nonsterile water for cleaning and preparing injectable medication in the CT suite. 

In summary, this epidemiologic investigation of 13 *S. maltophilia* BSIs did not find a point source for this outbreak but revealed intravenous contrast as a possible risk factor of concern. Although sampling of the 2 CT autoinjectors was negative for *S. maltophilia*, the facility implemented measures to decrease the risk for contamination from nonsterile water beyond what was recommended in the manufacturer’s IFU. Intensive investigations during outbreaks frequently do not identify a common source. However, addressing identified opportunities in infection control often results in a halt of transmission, and as of August 2025, no additional cases had been identified. Healthcare settings should discourage using nonsterile water for medical devices that could contact patient medications or care products to reduce the risk of contamination with waterborne pathogens such as *S. maltophilia*. 
